# Upregulation of microRNA-125b contributes to leukemogenesis and increases drug resistance in pediatric acute promyelocytic leukemia

**DOI:** 10.1186/1476-4598-10-108

**Published:** 2011-09-01

**Authors:** Hua Zhang, Xue-Qun Luo, Dan-Dan Feng, Xing-Ju Zhang, Jun Wu, Yu-Sheng Zheng, Xiao Chen, Ling Xu, Yue-Qin Chen

**Affiliations:** 1 Key Laboratory of Gene Engineering of the Ministry of Education, State Key Laboratory, for Biocontrol, Sun Yat-sen University, Guangzhou 510275, China; 2 The First Affiliated Hospital of Sun Yat-sen University, Guangzhou, 510080, China; 3 The Second Affiliated Hospital of Sun Yat-sen University, Guangzhou, 510120, China

**Keywords:** microRNA, pediatric acute promyelocytic leukemia (APL), treatment response, drug resistance

## Abstract

**Background:**

Although current chemotherapy regimens have remarkably improved the cure rate of pediatric acute promyelocytic leukemia (APL) over the past decade, more than 20% of patients still die of the disease, and the 5-year cumulative incidence of relapse is 17%. The precise gene pathways that exert critical control over the determination of cell lineage fate during the development of pediatric APL remain unclear.

**Methods:**

In this study, we analyzed *miR-125b *expression in 169 pediatric acute myelogenous leukemia (AML) samples including 76 APL samples before therapy and 38 APL samples after therapy. The effects of enforced expression of *miR-125b *were evaluated in leukemic cell and drug-resistant cell lines.

**Results:**

*miR-125b *is highly expressed in pediatric APL compared with other subtypes of AML and is correlated with treatment response, as well as relapse of pediatric APL. Our results further demonstrated that *miR-125b *could promote leukemic cell proliferation and inhibit cell apoptosis by regulating the expression of tumor suppressor BCL2-antagonist/killer 1 (Bak1). Remarkably, *miR-125b *was also found to be up-regulated in leukemic drug-resistant cells, and transfection of a *miR-125b *duplex into AML cells can increase their resistance to therapeutic drugs,

**Conclusions:**

These findings strongly indicate that *miR-125b *plays an important role in the development of pediatric APL at least partially mediated by repressing BAK1 protein expression and could be a potential therapeutic target for treating pediatric APL failure.

## Background

Pediatric acute promyelocytic leukemia (APL), which represents approximately 10% of pediatric AML cases, is a subgroup of acute myelogenous leukemia (AML) characterized by promyelocytic cell morphology (referred to as M3 in the French-American-British classification) [1-3]. APL is characterized by a specific t(15;17) translocation that encodes a fusion of the promyelocytic leukemia (PML) and retinoic acid receptor-α (RARA) proteins [1-5]. Although the outcomes for children and adults with APL have dramatically improved since the successful introduction of all-trans retinoic acid (ATRA) in combination with anthracycline-based chemotherapy, more than 20% of patients presenting with APL will die of the disease, and the 5-year cumulative incidence of relapse is 17% overall and more than 20% in children [6-8]. The combination of ATRA and chemotherapy as initial therapy has become an attractive strategy for all APL patients; unfortunately, approximately 10% of APL patients develop "retinoic acid syndrome (RAS)" [6,9,10]. Furthermore, the precise genes and pathways that exert critical control over the lineage fate during APL development remain unclear.

MicroRNAs (miRNAs), a novel class of small noncoding RNAs ranging from 19 to 25 nucleotides in size, regulate specific target genes through translational repression or direct mRNA degradation, thereby regulating many cellular functions, including cell proliferation, differentiation, and apoptosis [11-13]. Recent studies have shown that deregulated expression of specific miRNAs that modulate expression of oncogenes and tumor suppressors is associated with the development of malignancies and that specific miRNA expression signatures can be used to effectively classify human tumors [14]. MiRNA expression signatures associated with specific cytogenetic changes and clinical outcomes of adult CLL, AML, and Hodgkin's lymphoma have been reported [15-20]. Recent data suggest that miRNA inactivation by epigenetic mechanisms plays an important role in myelopoiesis and that modulating specific miRNA levels with drugs can lead to the downregulation of target oncogenes and restoration of cell differentiation [21]. Dysregulated miRNA expression in APL cells following retinoic acid (RA) induction was also reported [22-25]. These studies, however, mainly focused on APL cell lines and included very few clinical APL samples. The role of miRNAs in the clinical progression of APL, especially in pediatric APL, remains to be explained.

Our previous study found that *miR-125b *was up-regulated in pediatric primary AML using genome-wide miRNA expression profiles in 36 diagnostic acute leukemia bone marrow samples [26]. To further understand the role of *miR-125b *in pediatric AML, we analyzed *miR-125b *expression in 169 pediatric AML patients for whom clinical data were available. Interestingly, we found that *miR-125b *was reduced to normal levels in complete remission (CR) APL patients. Importantly, we found that *miR-125b *could promote proliferation and inhibit apoptosis of APL cells by targeting BCL2-antagonist/killer 1 (Bak1). The results imply that *miR-125b *functions as an oncogene in pediatric APL and that it has potential roles as a malignancy biomarker and a predictive marker of response to chemotherapy. In addition, we showed that transfection of the cells with *miR-125b *could increase cell resistance to chemotherapeutic drugs.

## Methods

### Patients, sample collection, and therapeutic methods

A total of 182 pediatric samples including 131 AML samples before therapy (including 76 PML-RARα-positive APL samples), 38 APL samples (PML-RARα-positive) after therapy and 13 normal samples from the First and Second Affiliated Hospital of Sun Yat-sen University and Beijing Children's Hospital were enrolled in this study. Patients' characteristics are available for all patients (see Table 1). Bone marrow was collected from patients by bone marrow puncture at diagnosis or at follow-up after therapy. The treatment protocol for AML patients is listed in Additional file 1. Table S1. Written informed consent for the biological studies was obtained from the parent/guardians. The study was approved by the Ethics Committee of the affiliated hospitals of Sun Yat-sen University.

**Table 1:** Pediatric AML patients' characteristics

AML Primary (N = 131)	Characteristics	Median (range)	No. (%)
	**Age at diagnosis, y**	7.8 (0-14)	
	**Sex**		
	Male		79 (60.3)
	Female		52 (39.7)
	**WBC count, ×10^9^/L**	18.2 (1.1-292)	
	Less than 10		76 (58)
	10-50		34 (26)
	50 or higher		21 (16)
	**FAB**		
	M1		9 (6.9)
	M2		27 (20.6)
	M3		76 (58)
	M4		5 (3.8)
	M5		11 (8.4)
	M6		3 (2.3)
**M3 after therapy (N = 38)**	#**CR**		33 (86.8)
	&**Relapse**		5 (13.2)

AML: acute myelogenous leukemia; WBC: white blood cell; FAB: French-American-British classification; CR: complete remission.

#: CR samples are from 33 patients of 76 M3 primary patients with three years clinical follow-up;

&: with three years clinical follow-up.

### Cell lines and cell cultures

Human leukemia cell line NB4, HL60, K562 and their drug resistant cell lines were maintained in RPMI 1640 medium containing 10% fetal bovine serum (Gibco BRL). Drug resistant cell lines included NB4-R1 (subclones of NB4 with ATRA resistance established by ATRA inducing progressively), NB4-R2 (subclones of NB4 with ATRA resistance established by mutating the E domain of PML/RARA), HL60/DOX (subclone of HL60 with doxorubicin-resistance established by drug inducing progressively) and K562/DOX (subclone of K562 with doxorubicin-resistance established by drug inducing progressively). 293 T cell line was maintained in DMEM medium containing 10% fetal bovine serum (Gibco BRL).

### Mouse model

Five-week-old BALB/c (nu/nu) mice were maintained under a specific pathogen-free condition in the Laboratory Animal Center, Sun Yat-sen University. All experiments were performed in accordance with our Institutional Animal Guidelines. The xenografted tumors were established by a single subcutaneous injection at the flank of ALB/c (nu/nu) mice with 2 × 10^6 ^HL60 cells (infected with lentivirus vectors that expressed miR-125b or miRNA negative control) in 0.1 ml RPMI-1640 medium. Each group consisted of at least 3 mice. The tumors were harvested, snap-frozen and stored at -80°C.

### RNA extraction and quantitative real-time polymerase chain reaction

Total RNA was isolated with Trizol (Invitrogen, Carlsbad, CA) according to the manufacturer's instructions. The following two quality criteria were used to assess total RNA quality. The first is purity. Both ratios, OD A260/A280 and OD A260/A230 were in the 1.8-2.1 region. The second is integrity. Total RNA quality has been assessed by denaturing gel electrophoresis electrophoresis and the ratio of 28S and 18S rRNA bands was approximately 2:1. Quantitative real-time RT-PCR (qRT-PCR) was performed as described [27] and employed a Hairpin-it™ miRNAs Real-Time PCR Quantization Kit containing stem-loop like RT primer, miRNA specific PCR primer and Molecular Beacon probe for *miR-125b *or the *U6 *RNA internal control (GenePharma, Shanghai, China). The expression of miR-125b relative to that in healthy samples was calculated using the 2-ΔΔCT method, and the expression of miR-125b relative to internal control U6 RNA in mouse was calculated using the 2-ΔCT method [28]. The efficiencies of PCR amplification per PCR-cycle (by a dilution curve) of both miR-125b and U6 from each patient are higher than 95% (Additional file 2. Figure S1).

### Statistical analysis

Fisher's exact test, paired t-test, and chi-square tests were used to compare baseline characteristics and average miRNA expression between different groups. All reported P values were two-sided and were obtained using SPSS software. P < 0.05 is considered significant.

### Target genes prediction

Target genes prediction was performed to meet the following two criteria. First, miRNA targets were analyzed using three algorithms, including TARGETSCAN http://www.targetscan.org, PICTAR http://pictar.mdc-berlin.de and miRBase http://microrna.sanger.ac.uk/sequences/index.shtml. Second, in order to reduce the number of false positives, only putative target genes predicted by at least two of the programs were accepted.

### Western blotting

Cells were homogenized in a lysis buffer (25 mM Tris-HCl (pH 7.4), 0.5 mM EDTA, 0.5 mM EGTA, 1 mM PMSF, 25 μg/ml leupeptin, 1 mM DTT, 0.5% Triton X-100) and centrifuged. Samples of supernatant, each containing 10 μg of total protein, were electrophoresed in a 5-15% gradient gel. Western blotting was performed as following. In brief, the proteins were transferred to polyvinylidene difluoride (PVDF) membranes. The membranes were blocked in 4% non-fat dry milk in Tris-buffered saline containing 0.1% Tween 20 (TBST) for 1 hr at room temperature and then probed overnight with rabbit polyclonal anti-Bak1 antibody (Sigma) in the same buffer. After three washes with TBST, the membranes were incubated for 1 hr with goat anti-rabbit IgG antibody (Sigma) conjugated to horseradish peroxidase (HRP). After washing with TBST, the membranes were exposed to a chemiluminescent reagent (ECL Plus, Amersham) for 1 min and then to Kodak X-ray film. Anti-β-actin antibody and anti-tublin antibody (Cell signaling technology) has been used to detect beta-actin and tublin.

### MiR-125b expression vector construct

Full-length double-stranded human pre-miR-125b, together with 305 bp of flanking sequence was amplified from human genomic DNA using PCR (primer information is available in Additional file 3. Table S2) and cloned into the pcDNA™6.2-GW/EmGFP-miR control vector (Invitrogen) to generate the *miR-125b *expression construct pcDNA-miR-125b. The PCR product was also cloned into pIRES2-EGFP (Invitrogen, Carlsbad, CA, USA) and the fragment containing CMV promoter and *miR-125b *precursor was then cloned into pINCO retroviral vector (Genechem, Shanghai, China) to generate lv-miR-125b. Insertions were verified by DNA sequencing.

### pGL3-BAK1 reporter construct and mutagenesis

The 252 bp 3'-untranslated terminal region (UTR) segment of Bak1 was amplified by PCR from human genomic DNA and inserted into the pGL3-control vector (Promega) using the XbaI site immediately downstream from the stop codon of luciferase for *miR-125b *functional analysis. Similarly, recombinant pGL3 reporter vectors for other selected possible targets were constructed. The Bak1 mutant, pGL3-BAK1-M, which had 8 bp deleted from the site of perfect complementarity to *miR-125b*, was generated using PCR deletion Wild-type and mutant insertions were confirmed by DNA sequencing. Primer information is available in Additional file 3. Table S2.

### Luciferase assay

0.2 μg of pcDNA-miR-125b or pcDNA-control was cotransfected to 293 T cells with 0.1 μg of pGL3-control or recombinant pGL3 reporter vector and 0.1 μg of the control vector pRL-TK containing Renilla luciferase and TK promoter (Promega) using lipofectamine 2000 (Invitrogen). For NB4 cells, 5 μg of pcDNA-miR-125b or pcDNA-control, 5 μg of pGL3-control or pGL3-BAK1 or pGL3-BAK1-M Firefly luciferase report vector and 2.5 μg of the control vector pRL-TK were cotransfected using electroporation. 293 T cells or NB4 cells were grown in 24-well plates (1 × 10^5 ^cells/well) in 10% FBS in DMEM or RPMI-1640 supplemented with sodium pyruvate at 37°C in a humidified atmosphere of 5% CO_2_. Firefly and Renilla luciferase activities were measured consecutively using dual-luciferase assays (Promega) 24 hrs after transfection, according to the manufacturers' instructions.

### Cell proliferation and apoptosis assay

NB4 cells, HL60 cells, NB4-R1 cells, NB4-R2 cells and HL60/DOX cells (1 × 10^4 ^per well) were plated in 96-well plates in RPMI medium 1640 and 10% FBS that was supplemented with sodium pyruvate at 37°C in a humidified atmosphere of 5% CO_2_. Cells were transfected with 100 nM *miR-125b *duplex (Ambion), scrambled duplex (negative control, Ambion), or 200 nM *miR-125b *antisense (Ambion) using lipofectamine 2000 (Invitrogen). After culturing for 1, 2 or 3 days, the MTT assay (Promega) or CCK-8 assay (Dojindo Molecular Technologies) was carried out to analyze cell proliferation. For apoptosis, forty-eight hours following the transfection, cells were labeled with Annexin V/PI (MultiSciences Biotech) and analyzed by flow cytometry.

### Cell cytotoxicity assay

HL60 or HL60/DOX cells were seeded in 24-well plates at a density of 3 × 10^5 ^cells per well and transfected with 100 pmol scrambled duplex, *miR-125b *duplex or *miR-125b *antisense, or scrambled duplex (Ambion) in three independent replicates, using Nenon transfection equipment (Invitrogen) according to the manufacturer's protocol with program settings of 1400 V, 10 ms width, and 3 pulses. Forty-eight hours after transfection, cells were reseeded in 96-well plates at a density of 1.5 × 10^4 ^per well in the presence of *miR-125b *and treated with doxorubicin at a range of concentrations (5 to 25 μg/ml) in medium for 72 hrs. Cell survival was analyzed using the Cell Titer 96 Aqueous One Solution Cell Proliferation Assay (Promega).

## Results

### miR-125b is highly expressed in pediatric APL

**Figure 1:**
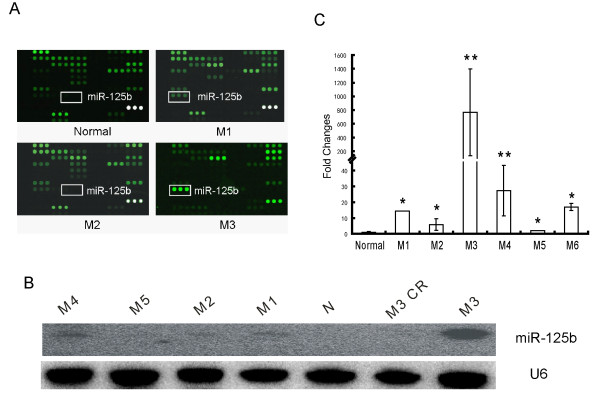
**Differential expression of miR-125b in pediatric AML patients**. (A) Differential expression of miR-125b in normal samples and different subtypes of pediatric AML determined by miRNA microarray analysis (the same part of different microarray chips is shown). Bright green dots indicate highly expressed miRNAs. (B) Expression of miR-125b in the normal pool (N); different subtype pools of pediatric AML and M3 complete remission pool (M3 CR) were validated by northern blot. (C) Expression levels of miR-125b in subtypes of 131 pediatric AML were analyzed with qRT-PCR. Data are presented as the fold change of miR-125b expression in patient samples with respect to expression in bone marrow mononuclear cells (MNC) from 13 healthy donors. The average miR-125b expression of each subtype was statistically compared with the average normal value. *p < 0.05; **p < 0.01.

Using genome-wide miRNA expression profiles in 36 diagnostic acute leukemia bone marrow samples, we previously identified 17 up-regulated and 18 down-regulated miRNAs that were significantly deregulated in pediatric primary AML compared with healthy samples [26]. Remarkably, *miR-125b *was significantly up-regulated in pediatric APL patients compared with other AML subtypes (Figure 1A). This result was further confirmed by northern blot (Figure 1B). This finding raised the possibility that *miR-125b *might play an important role in the development of pediatric APL.

**Figure 2:**
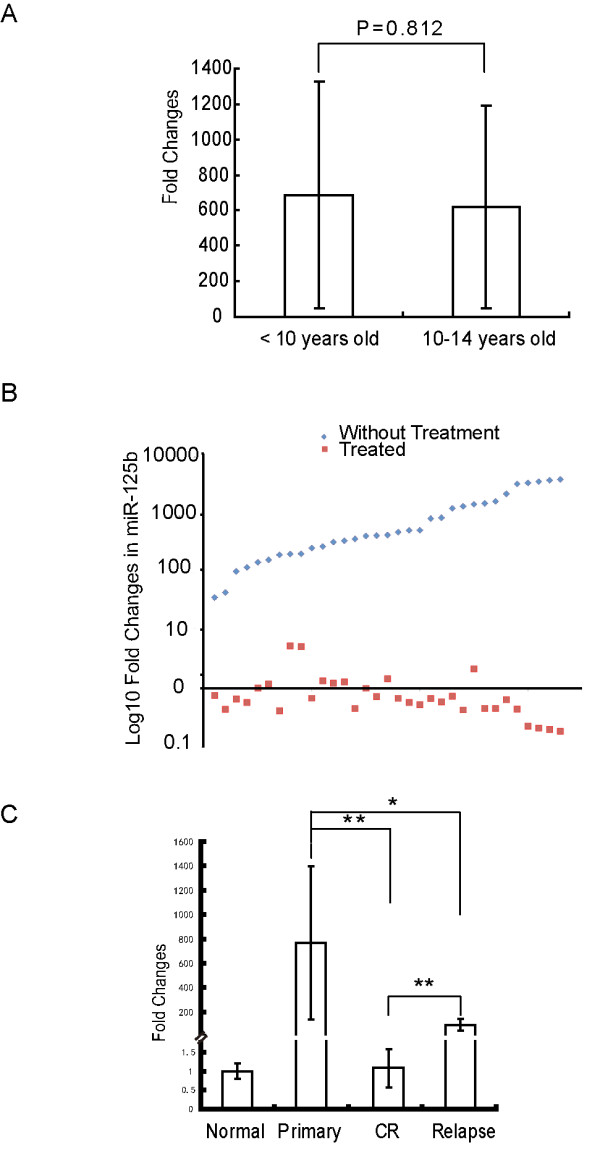
**Expression of miR-125b varied in different therapeutic response groups of pediatric APL**. The qRT-PCR assay was repeated three times, and similar results were obtained. Representative results are shown as means ± standard deviation (M ± SD). Data presented are the fold changes of expression with respect to the bone marrow MNC from five healthy donors. (A) Expression level of miR-125b in different age groups of pediatric APL patients. (B) The expression levels of miR-125b in 33 pediatric APL patients before and after therapy are presented as fold changes of expression with respect to expression in bone marrow mononuclear cells (MNC) from healthy donors. MiR-125b levels for the same patient before and after therapy were paired. Data were sorted from lowest to highest levels of miR-125b in non-treated patients. (C) Expression level of miR-125b in pediatric APL patients before and after therapy. *p < 0.05, **p < 0.01.

To clarify the differential expression pattern of *miR-125b*, we performed large-scale qRT-PCR assays on bone marrow samples from 131 pediatric primary AML samples, including 76 APL patients with PML-RARα-positive APL and 55 patients with other subtypes (M1, M2, M4, M5, M6). As shown in Figure 1C, *miR-125b *showed exceptionally high expression in APL (average of 760-fold higher than the normal population), although *miR-125b *expression in other subtypes was also higher than normal. However, the expression of *miR-125b *was not significantly different between younger (less than 10 years of age) and older (between 10 and 14 years of age) pediatric patients (Figure 2A). Our results suggested that *miR-125b *might be involved in APL pathogenesis and that it might be used as a biomarker for malignancy in pediatric APL.

### Induction therapy suppresses miR-125b expression in pediatric APL

All pediatric APL (M3 subtype) patients received a substantially distinct therapy utilizing the modified PETHEMA protocol (Additional file 1. Table S1) after diagnosis. We then collected 33 matched-pair APL samples at diagnosis and complete remission (CR) with PML-RARα-positive and with three years of clinical follow-up. We also collected 5 relapsed patients for comparison. To establish the relevance of *miR-125b *expression before and after therapy treatment, we investigated *miR-125b *expression in pairs of samples with diagnosis-CR or diagnosis-relapse. The results showed that in all CR patients, *miR-125b *expression decreased sharply after therapy to the same levels as in the normal controls (Figure 2B), which may be due to largely reduced leukemic cells after therapy. However, the expression level in the relapse patients was higher than normal controls, although induction therapy suppressed *miR-125b *expression in these patients to some degree (Figure 2C). The significantly different expression of *miR-125b *between pediatric primary, CR and relapse patients suggested that *miR-125b *could be a biomarker for clinical outcome. This possibility will be further studied using a larger number of patients. This finding raised the possibility that *miR-125b*, which is involved in the timing of tissue development and cell differentiation [29], might function as an oncogene in pediatric APL.

### miR-125b represses endogenous Bak1 protein in myeloid cell lines and pediatric APL samples and is associated with disease development and treatment outcomes

**Figure 3:**
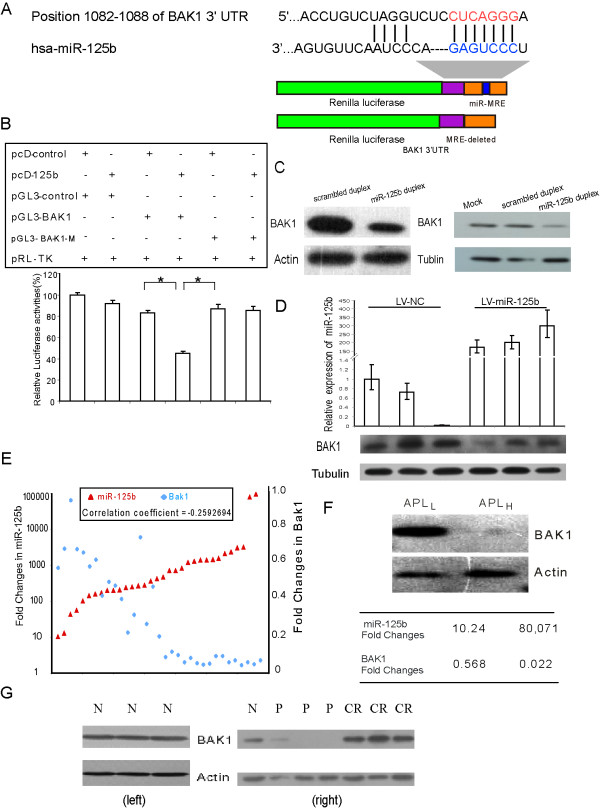
**Exogenous miR-125b deregulates Bak1 protein**. (A) An outline of luciferase reporter assay for validating the interaction of miR-125b with the 3' UTR of BAK1 is shown. Red text indicates the ''seed'' regions. In mutant reporter constructs, the MRE was deleted. (B) Repression of luciferase activity due to the interaction between miR-125b and the predicted MREs in the luciferase-Bak1-3' UTR constructs. The values represent the average ± SD (n = 3). *p < 0.05. (C) Western blot analysis of BAK1 expression in NB4 cells (left panel) or HL60 cells (right panel) after transfection with 100 nM miR-125b duplex or scrambled duplex. (D) The effects of suppression of miR-125b on Bak1 in mouse model. Upper: the overexpression of miR-125b was examined using qRT-PCR; lower: western blot analysis showed the Bak1 protein was repressed by miR-125b. lv-miR-125b: lentivirus vectors that expressed miR-125b; lv-NC: lentivirus vectors that expressed miR-NC, miRNA negative control. (E) Bak1 protein expression was inversely correlated with miR-125b levels in 33 of 47 pediatric APL patient samples. Red triangle: miR-125b expression (fold change vs. normal average); light blue diamond: Bak1 protein expression (fold change vs. normal average). Both miR-125b and Bak1 average expression levels of healthy donors were set at 1. (F) Western blotting images showing typical high and low Bak1 protein expression levels. The Bak1 protein level was quantified from western blot bands normalized to the β-actin level. The average expressions of Bak1 and miR-125b, presented as fold change compared with healthy samples, are listed in the table. (G) Bak1 protein expression in three normal donors using western blot (left), selected typical Bak1 protein expression in normal donor and pediatric patients with different ATRA responses was analyzed by western blot (right). N, normal donor; P, samples collected in primary diagnosis without treatment; CR, complete remission after therapy.

Elucidating the targets of miRNAs is still a major part of the miRNA functional investigation. We selected 15 potential targets that contain the conserved miRNA response elements (MREs) of *miR-125b *in their 3' UTR and had been reported to be related to leukemogenesis for further experimental investigation. Among them, we confirmed that four putative targets (BAK1, MAP3K10, MCL1, and TRIAP1) were repressed by *miR-125b *when transfected to 293T cells, with a repression rate of more than 25%, as measured by a luciferase assay (Additional file 4. Figure S2A). Remarkably, we found that the tumor suppressor Bak1 had the most reduced activity, which suggested that Bak1 was likely to be an important target regulated by *miR-125b *in leukemia. To further test our hypothesis, we constructed a recombinant pGL3-BAK1-M with a mutant *miR-125b *binding site (Figure 3A). pGL3-BAK1 and pGL3-BAK1-M were then cotransfected into NB4 cells either with or without pcDNA-miR-125b using the electroporation method. As shown in Figure 3B, transfection of *miR-125b *reduced luciferase activity in pGL3-BAK1 transfected NB4 cells by about 40%. However, in NB4 cells transfected with either wild-type or mutant reporter in the absence of pcDNA-miR-125b, there was almost no change in luciferase activity. This finding demonstrates that Bak1 is a target of *miR-125b *in leukemic cells and that the putative *miR-125b *binding site is critical for *miR-125b *regulation of Bak1 expression.

To further investigate whether overexpression of *miR-125b *can promote APL myeloid cell progression by targeting tumor suppressor Bak1, we transfected two APL cell lines (NB4 and HL60) with 100 nM *miR-125b *duplex using the electroporation method (Additional file 4. Figure S2B). Bak1 expression was strongly reduced compared with that transfected with scrambled duplex in both myeloid cell lines NB4 (Figure 3C, left panel) and HL60 (Figure 3C, right panel). To uncover the effects of suppression in vivo, HL60 cells were infected with lentivirus vectors that expressed miR-125b (lv-miR-125b) or miRNA negative control (lv-NC), and then transplanted into nude mice with SC injection. Both groups of mice transplanted with lv-miR-125b-HL60 cells and lv-NC-HL60 cells developed solid tumors three weeks later. The overexpression of miR-125b was examined using qRT-PCR (Figure 3D, upper). The western blot analysis showed the Bak1 protein was repressed in the lv-miR-125b-HL60 tumors compared with the lv-NC-HL60 tumors transplanted with miRNA negative control (Figure 3D, lower).

Next, to examine the possibility that Bak1 was also regulated by *miR-125b *in clinical samples, we measured the expression levels of these two molecules in 47 pediatric APL clinical samples (only 47 samples have enough proteins to be used for western blot ananlysis) and five normal donors. We found that the levels of Bak1 and *miR-125b *were inversely correlated in 70.2% (33/47) of the pediatric APL samples (Figure 3E). For comparison, Figure 3F illustrates typical Bak1 protein expression in the 33 samples with the highest (APL_H_) and lowest (APL_L_) levels of expressed *miR-125b*. The levels of *miR-125b *expression for APL_H _and APL_L _were 80,071-fold and 10.24-fold higher than the normal average, respectively, while the levels of Bak1 expression were 50-fold lower in APL_H _and 2-fold lower in APL_L_. These results strongly suggest that Bak1 is a target of *miR-125b *in pediatric APL.

Because induction therapy suppressed *miR-125b *expression in pediatric APL patients, we would expect that, as a target of *miR-125b*, Bak1 levels would also be affected by therapeutics. We therefore analyzed the levels of Bak1 and *miR-125b *in samples from follow-up patients and compared the profiles with the profiles obtained from the same patients before therapy. In addition, three normal marrows were used as controls. Bak1 expression was increased in about 60% of CR patients (19/33) receiving therapeutics (Figure 3G). Thus, a concordant negative correlation existed between *miR-125b *levels and Bak1 levels with respect to disease development and treatment outcome.

### Exogenous miR-125b promotes leukemic cell proliferation and inhibits apoptosis of leukemic cells

**Figure 4:**
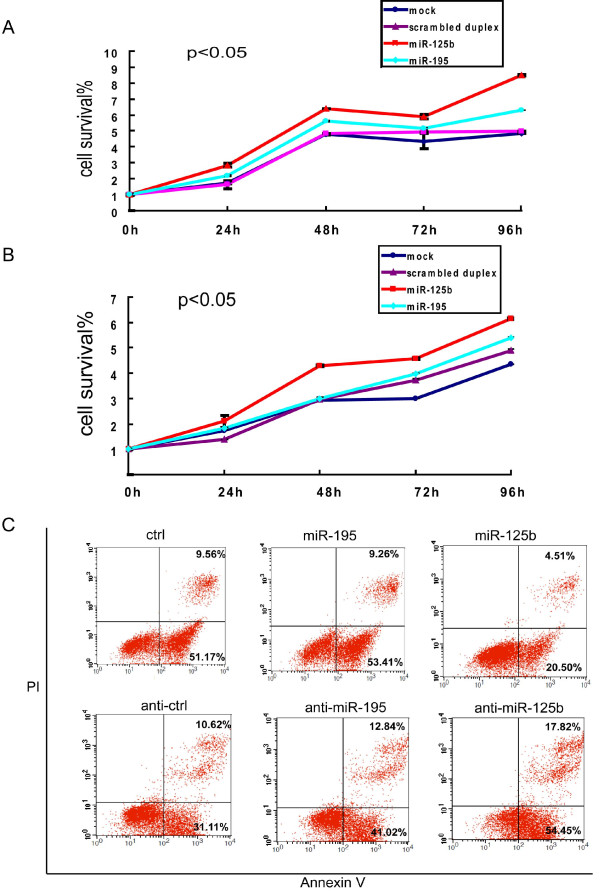
**miR-125b promotes cell proliferation and inhibits apoptosis in leukemic cell lines NB4 and HL60**. (A) Time-dependent effects of miR-125b on NB4 cell proliferation were confirmed using an MTT assay. (B) Time-dependent effects of miR-125b on HL60 cell proliferation were confirmed using an MTT assay. At least three independent experiments were performed and similar results were obtained. Data are represented as means ± SD from 3 independent experiments. P < 0.05, compared with mock, scrambled duplex and miR-195 at 48, 72 and 96 hrs. (C) Apoptosis in a leukemic cell line transfected with miR-125b duplex, as detected by flow cytometry. Upper panel: The NB4 cells were transfected with 100 nM scrambled duplex, miR-195 or miR-125b duplex, respectively. Lower panel: The NB4 cells were transfected with 100 nM antisense control, miR-195 or miR-125b antisense, respectively. Forty-four hours following transfection, camptothecin was added to induce cells for four hours and then cells were labeled with Annexin V/PI and analyzed by flow cytometry. Three independent experiments were performed and similar results were obtained.

Bak1 is a pro-death protein and can promote programmed cell death [30]. Overexpression of *miR-125b*, which inhibits Bak1 expression, may therefore result in reduced apoptosis and enhanced proliferation of leukemic cells and further induce oncogenesis. Thus, we measured cell proliferation and viability of transfected NB4 cells using an MTT assay and CCK-8 assay. As expected, proliferation of NB4 cells was stimulated by the transfected *miR-125b *duplex (Figure 4A) or lv-miR-125b (Additional file 5. Figure S3), and the effect was time-dependent. In addition, transfection of HL60 cells with 100 nM *miR-125b *promoted cell proliferation (Figure 4B). These results showed that *miR-125b *may function as an oncogene and may contribute to APL progression.

We then transfected the myeloid leukemic cell line NB4 with *miR-125b *duplex (mimics) and *miR-125b *inhibitor (anti-miR-125b) to assess the possible role of *miR-125b *in apoptosis in leukemic cells. However, in cancer cells, p53 and Bak1 protein are mainly localized in the cytoplasm, so the endogenous activity of Bak1 is usually insufficient to modulate apoptosis [31]. Additionally, a previous study showed that ectopic expression of *miR-125b *in cells only suppresses apoptosis when the mitochondrial pathway is fully activated by exposure to the drug [32]. We then treated the cells with 7-ethyl-10-hydrol-camptothecin and performed flow cytometry assays after 48 hrs of transfection. The results showed that ectopic expression of *miR-125b *significantly suppressed 7-ethyl-10-hydrol-camptothecin-induced apoptosis of NB4 cells (Annexin V-FITC positive) compared with control and *miR-195 *duplex (Figure 4C, upper panel).

The ability of transfection with *miR-125b *antisense to control apoptosis was also examined. Interestingly, after being transfected with 100 nM *miR-125b *(antisense), the percentage of 7-ethyl-10-hydrol-camptothecin-induced apoptotic NB4 cells (ANNEXIN V-FITC positive) at 48 hrs increased compared with control and *miR-195 *duplex (Figure 4C, lower panel). Similar results were obtained in HL60 cells (Additional file 6. Figure S4A). These results demonstrate that transfection of *miR-125b *antisense can remarkably increase cell apoptosis in leukemia cells. Furthermore, we inferred that *miR-125b *directly regulates Bak1 expression, thereby modulating the susceptibility of leukemic cells to apoptosis.

**Figure 5:**
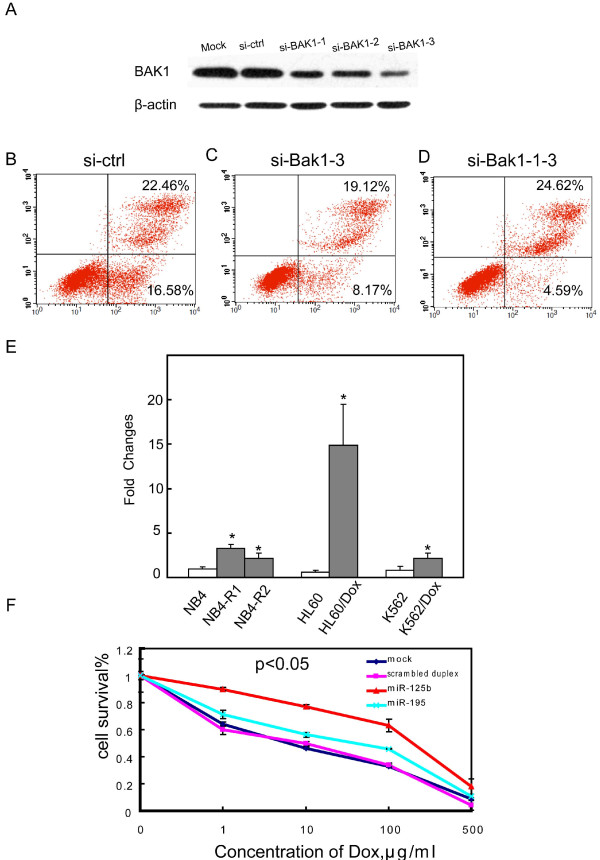
**Apoptosis after knockdown of Bak1 and the involvement of miR-125b in drug-resistant leukemic cells**. (A) Western blotting images showing low Bak1 protein expression when NB4 cells were transfected with three different strands of si-RNA of Bak1. (B), (C) and (D) The NB4 cells were transfected with 100 nM si-RNA control, a strand of si-Bak1-3 or a mixture of these three strands of si-Bak. Forty-eight hours following transfection, camptothecin was added and cells were labeled with Annexin V/PI and analyzed by flow cytometry. Three independent experiments were performed and similar results were obtained. (E) miR-125b expression was up-regulated in drug-resistant cell lines compared to their drug-sensitive parental cells. *p < 0.05 compared with corresponding parents cells. (F) Transfection of HL60/DOX cells with miR-125b duplex increases their resistance to DOX treatment. The IC_50 _of HL60/DOX cells transfected with miR-125b duplex was higher (p < 0.05) compared with the IC_50 _for HL60/DOX cells transfected with miRNA duplex control and miR-195. Three independent experiments were performed.

To prove the physiological relevance of Bak1, RNAi against Bak1 was performed to analyze its role in myeloid cell apoptosis. The transfection of NB4 cells with different pieces of siRNAs against the Bak1 mRNA resulted in greatly decreased levels of the endogenous protein Bak1, especially for the pieces si-Bak1-1, si-Bak1-2 and si-Bak1-3 (Figure 5A). To test if down-regulation of Bak1 was able to inhibit myeloid cell apoptosis, we detected cell apoptosis 48 hrs after transfection of NB4 cells with si-Bak1-3 and a mixture of the three strands of siRNA. The number of apoptotic leukemic cells was obviously reduced by the subsequent downregulation of Bak1 (Figure 5B-D). Altogether, these data indicate that the artificial increase of *miR-125b *or reduction of Bak1 levels was able to inhibit myeloid leukemic cell apoptosis.

### Up-regulation of miR-125b levels was found in resistant cells and reduction of the expression levels increased their sensitivity to therapeutic drugs

We noted above that the expression of *miR-125b *in all CR patients normalized after therapy, while it remained much higher in relapse patients (Figure 2C). We also found that *miR-125b *expression was slightly up-regulated in four drug-resistant cell lines, including HL60/DOX, K562/DOX (subclone of K562 with doxorubicin-resistance) and NB4-R1, as well as NB4-R2 (subclones of NB4 with ATRA resistance), when compared with their drug-sensitive parental cells (Figure 5E). These findings imply that *miR-125b *contributes to the drug-resistant nature of leukemic cells and that up-regulation of *miR-125b *levels in cells may increase their resistance to therapeutic drugs.

To address this issue, we transfected drug-resistant HL60/DOX cells and NB4-R1 with *miR-125b *duplex. Transfection of HL60/DOX cells with *miR-125b *duplex increased their resistance to DOX treatment. The IC50 of HL60/DOX cells transfected with *miR-125b *duplex was higher (p < 0.05) compared with the IC50 for HL60/DOX cells transfected with the miRNA duplex control and *miR-195 *duplex (Figure 5F). We also reproduced the same experiment in NB4-R1, and the results are shown in Additional file 6. Figure S4B. Taken together, the above results indicate a link between *miR-125b *dysregulation and drug resistance. However, the precise pathway and mechanism of action of *miR-125b *in the acquisition of drug resistance remains elusive.

## Discussion

In our previous investigation, we analyzed genome-wide miRNA expression profiles in pediatric AML and identified miRNA patterns specific to this disease. We also found that unique miRNA expression profiles were present in different subtypes of AML [26]. These results imply that particular miRNAs might have specific roles in carcinogenesis or in the development of certain subtypes of leukemia. We noted that the expression of some miRNAs, including *miR-125*, was strikingly higher in pediatric APL than other subtypes of AML. This phenomenon led us to consider whether these miRNAs are oncogenic, whether they cause specific subtypes of pediatric leukemia or simply accompany them, or, alternatively, whether they represent a tumor suppressor feedback mechanism that is superseded by malignancy. Elucidating these questions should enhance our understanding of the malignant progression of pediatric APL. Therefore, to address these questions, we focused on the role of highly up-regulated *miR-125b *in pediatric APL.

The expression of *miR-125b *has been investigated in many human cancers. It is down-regulated in human breast cancer, thyroid anaplastic carcinomas, ovarian cancer, squamous cell carcinoma of the tongue, and hepatocellular carcinoma, suggesting that it functions as a tumor suppressor [33-38]. Myeloid cell differentiation arrest by *miR-125b *was also found in leukemia. M. Bousquet et al. demonstrated that *miR-125b *was able to interfere with primary human CD34+ cell differentiation and overexpression of *miR-125b *is sufficient both to shorten the latency of BCR-ABL-induced leukemia and to independently induce leukemia in a mouse model [39,40]. These results reveal that *miR-125b *is important in cancer development.

The most important finding of this study was the discovery that *miR-125b *levels are related to the treatment response in pediatric APL patients. In patients with APL and positive PML-RARα gene fusion, *miR-125b *expression was much higher than in patients with other subtypes of AML, suggesting that *miR-125b *may be involved in *PML/RARA-*positive pediatric APL patients. However, the detailed mechanism of its role remains to be characterized. Notably, we found that *miR-125b *expression is associated with both disease development and treatment response. These results thus suggest a potential that *miR-125b *may represent a suitable biomarker for predicting the effect of chemotherapy.

Shi et al. showed that in prostate cancer, upregulated *miR-125b *could promote proliferation and targets Bak1 [40,41]. Zhou et al. demonstrated that Bak1 is a direct target of *miR-125b *in breast cancer [42]. However, the role of *miR-125b *in leukemia, especially in pediatric APL, remains unclear. We also found an inverse correlation between levels of *miR-125b *and Bak1 and obtained strong evidence that *miR-125b *could directly inhibit Bak1 expression in pediatric APL. Taken together, these findings suggest that up-regulated *miR-125b *may be part of a common regulatory pathway in different cancers. Furthermore, exogenous *miR-125b *is capable of promoting proliferation of malignant cells. This finding indicates that *miR-125b *may function as an oncogene by inhibiting APL cell apoptosis and promoting APL cell proliferation.

Although conventional therapy, such as the modified PETHEMALPA99 protocol, succeeded in obtaining long term CR in more than 70% of APL patients, early death and relapse in some patients are still a major concern in therapeutics, especially for pediatric APL. However, the mechanism of the patients' resistance to chemotherapeutic treatment is not entirely clear. Therefore, distinguishing the reason behind drug resistance and developing biomarkers are critical.

We have shown that *miR-125b *differentially expressed between pediatric APL primary, CR and relapse patients, suggesting a potential that high *miR-125b *expression might be used as a biomarker to indicate the treatment response of pediatric APL patients, although the detailed mechanism is still unknown. More importantly, transfection of NB4 and HL60 cells with *miR-125b *effectively inhibited apoptosis of these leukemic cells. Previously, M. Bousquet et al. reported that *miR-125b *was able to block the differentiation of NB4 cells induced by ATRA [39]. Recently, *miR-125b *was also found associated with drug resistance in breast cancer and pediatric acute lymphoblastic leukemia [42,43]. In this study, we also demonstrated that up-regulation of *miR-125b *in pediatric APL cells can increase their resistance to therapeutic drugs. We speculated that increased *miR-125b *expression might block the differentiation of hematopoietic precursors and respond to induction by ATRA or other chemotherapeutic drugs.

The expression of *miR-125b *both in pediatric and adult leukemia is currently not well understood. In adult APL, M. Bousquet et al. showed that myelodysplastic syndrome and AML patients carrying the t(2;11)(p21;q23) translocation were associated with *miR-125b *up-regulation [39]; however, they only studied 19 adult leukemia patients carrying the t(2;11)(p21;q23) translocation and did not determine whether the expression of *miR-125b *was up-regulated in adult APL patients. Another miRNA expression profiling assay suggested that *miR-125b *was up-regulated in seven adult APL patients, but this was not validated [44]. Thus, *miR-125b *expression should be further examined on a large scale with adult APL patients. Remarkably, in our large-scale qRT-PCR assay using 131 pediatric primary AML samples (M1 to M6), we found that *miR-125b *was exceptionally highly expressed in the pediatric APL subtype compared with other AML subtypes. Because pediatric AML is a heterogeneous disease made up of various leukemia subtypes that differ markedly in their cytogenetics, the identification of these specific cytogenetic characteristics within each subtype will be helpful for elucidating the mechanism of oncogenesis in particular AML subtypes. Furthermore, this information may benefit the design of chemotherapeutic strategies for patients.

In conclusion, this is the first study of *miR-125b *expression in pediatric APL patients. Our results suggest that *miR-125b *might be used as a biomarker of malignancy and as a biomarker to evaluate the effectiveness of chemotherapy in pediatric APL. We also found that *miR-125b *promots proliferation and inhibits cell apoptosis at least partially mediated by targeting tumor suppressor Bak1 in pediatric APL. More importantly, forcing expression of *miR-125b *can increase resistance to therapeutic drugs, suggesting that *miR-125b *is a potential therapeutic target for pediatric APL failure.

## Competing interests

The authors declare that they have no competing interests.

## Authors' contributions

HZ, and XQL contributed equally to this work, performing experiments, analyzing the data, and writing the manuscript; DDF, YSZ, XJZ and JW performed and analyzed western blot and qRT-PCR data; ZGL, XC, and ZYK provided patient samples and clinical data and designed the experiments; LX and YQC designed experiments and edited the manuscript. All authors critically read and approved the final manuscript.

## Supplementary Material

Additional file 1 **Table S1. AML treatment protocol**. Click here for file

Additional file 2 **Figure S1. The efficiencies of PCR amplification of miR-125b and U6**. The efficiencies of PCR amplification of both miR-125b (A) and U6 (B) from the same patient are higher than 95%. A ten-fold dilution of total RNA was used from 100 ng to 0.01 ng. Click here for file

Additional file 3 **Table S2. Primers information**. Click here for file

Additional file 4 **Figure S2. Luciferase reporter assay analyzed the targets of miR-125b and miR-125b expression level in HL60 cells after transfection using the electroporation method**. (A) Luciferase activity was decreased because the 3' UTR of BAK1, MAP3K, MCL1 and TRIAP1 was binding to miR-125b. (B) miR-125b expression level in HL60 cells was measured by qRT-PCR analysis after transfection with miRNA scrambled duplex, miR-125b mimics, miRNA inhibitor negative control and miR-125b inhibitor. Error bars represent standard deviation and were obtained from three independent experiments. Click here for file

Additional file 5 **Figure S3. miR-125b promotes cell proliferation in NB4**. (A) The qRT-PCR assay was used to measure expression profiles of miR-125b. Data presented are the fold changes of miR-125b in NB4 cells transduced with lv-miR-125b and lv-Src; (B) Cell proliferation was detected using CCK-8 assay. NB4, NB4-miR-125b and NB4-Scr cells with different expression profiles of miR-125b were induced by 1 uM ATRA and cell proliferation was measured at 24 hrs, 48 hrs and 72 hrs. *p < 0.05 compared with mock (NB4) and NB4-Scr. Click here for file

Additional file 6 **Figure S4. miR-125b inhibits apoptosis in HL60 cells and increases NB4-R1 cells resistance to ATRA treatment**. (A) The HL60 cells were transfected with 100 nM antisense control or miR-125b antisense, respectively. Forty-four hours following transfection, camptothecin was added to induce cells for four hours and then cells were labeled with Annexin V/PI and analyzed by flow cytometry. Three independent experiments were performed and similar results were obtained. (B) Transfection of NB4-R1 cells with miR-125b duplex increases their resistance to ATRA treatment. Three independent experiments were performed. Click here for file
