# Supplementary material for: Upregulation of microRNA-125b contributes to leukemogenesis and increases drug resistance in pediatric acute promyelocytic leukemia
Source: Mol Cancer. 2011 Sep 1;10:108. doi: 10.1186/1476-4598-10-108 (PMC3189170; doi:10.1186/1476-4598-10-108)
Supplement: Additional file 1 — Table S1. AML treatment protocol. [file 1476-4598-10-108-S1.DOC]

**Table S1. AML treatment protocol**

**GZCLG－AML2004 Modified PETHEMA LPA99**

**(For non FAB-M3) (For FAB-M3)**

**Induction**

NVT 10mg/m2 IV d1-3 ATRA 25mg/m2 PO d1 to CR

Ara-C 75mg/m2 SC q12h d1-7 NVT 10mg/ m2 IV d3-5

VP16 150mg/m2 IV d5-7

**Consolidation**

Course 1 Ara-C 2g/m2 IV q12h d1-3 1st month ATRA 25mg/m2 PO d1-15

NVT 10mg/m2 IV d2-3 NVT 10mg/m2 IV d1-3

Course 2 Ara-C 2.5g/m2 IV q12h d1-3 2nd month ATRA 25mg/m2 PO d1-15

VP16 150mg/m2 IV d2-3 DNR 40mg/m2 IV d1-3

Course 3 Ara-C 2g/m2 IV q12h d1-3 3rd month ATRA 25mg/m2 PO d1-15

NVT 10mg/m2 IV d2-3 NVT 10mg/m2 IV d1-3

Course 4 Ara-C 2g/m2 IV q12h d1-3

NVT 10mg/m2 IV d2-3

**Maintenance**

MTX 20mg/m2 PO qw

6mp 50mg/m2 PO qn

ATRA 25 mg/m2 PO d1-15 every 3

months

The duration of maintenance is 2 years

PO indicates orally; IV, intravenous; SC, subcutaneous injection.

CR:complete remission.
