# Supplementary figures and images for: Upregulation of microRNA-125b contributes to leukemogenesis and increases drug resistance in pediatric acute promyelocytic leukemia
Source: Mol Cancer. 2011 Sep 1;10:108. doi: 10.1186/1476-4598-10-108 (PMC3189170; doi:10.1186/1476-4598-10-108)

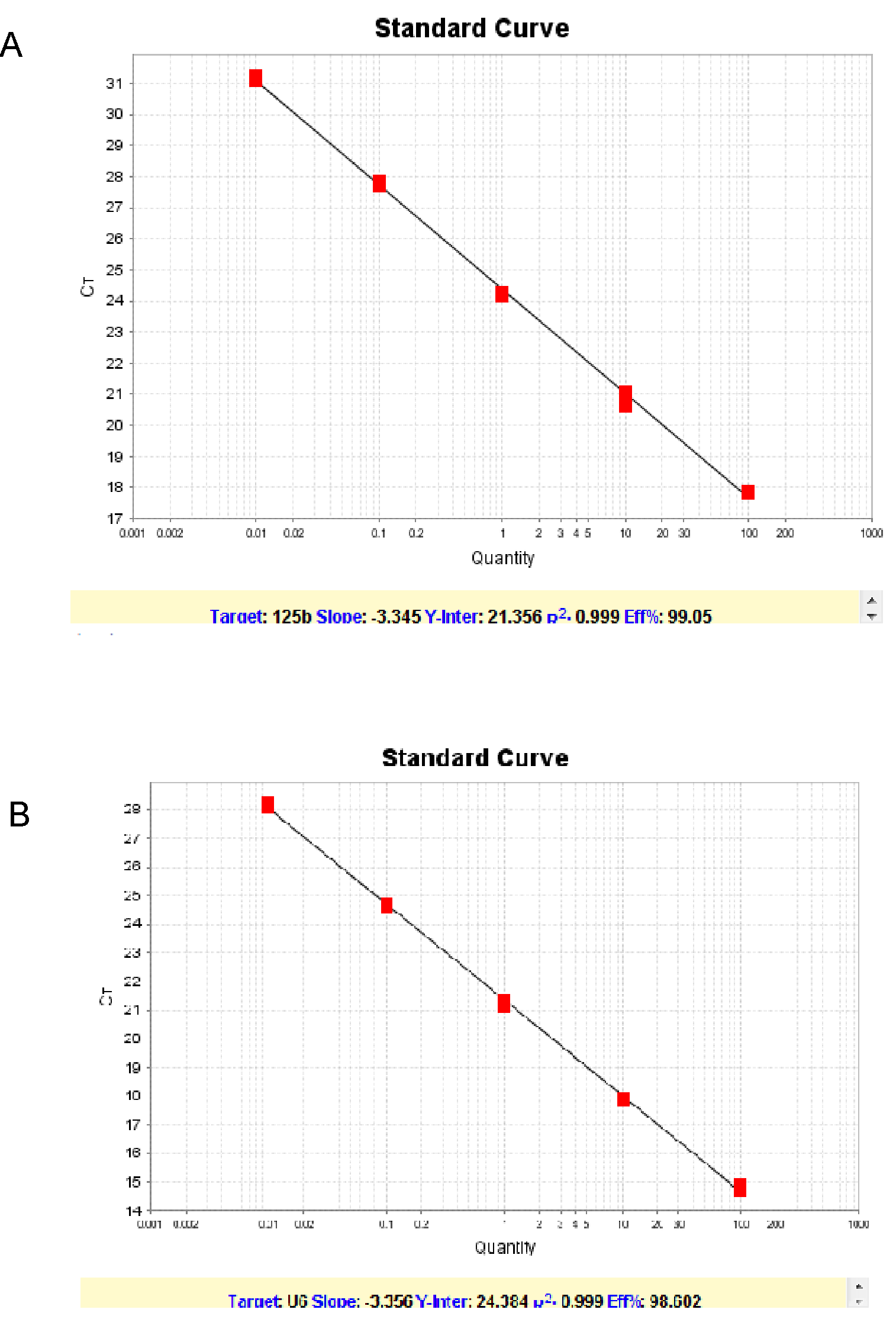

Supplement: Additional file 2 — Figure S1. The efficiencies of PCR amplification of miR-125b and U6. The efficiencies of PCR amplification of both miR-125b (A) and U6 (B) from the same patient are higher than 95%. A ten-fold dilution of total RNA was used from 100 ng to 0.01 ng. [file 1476-4598-10-108-S2.TIFF]

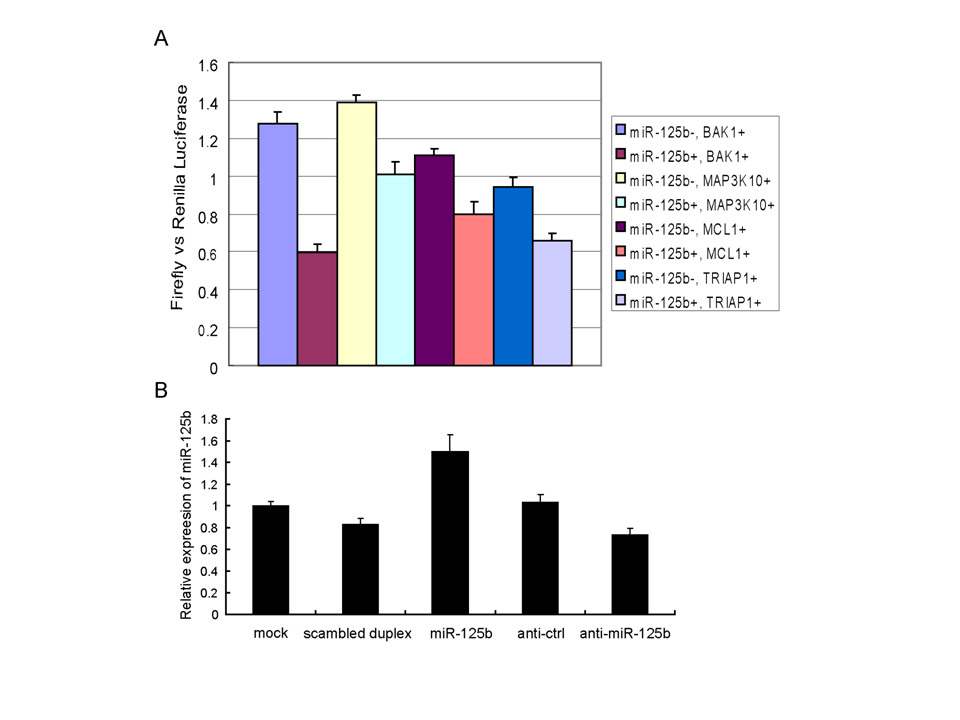

Supplement: Additional file 4 — Figure S2. Luciferase reporter assay analyzed the targets of miR-125b and miR-125b expression level in HL60 cells after transfection using the electroporation method. (A) Luciferase activity was decreased because the 3' UTR of BAK1, MAP3K, MCL1 and TRIAP1 was binding to miR-125b. (B) miR-125b expression level in HL60 cells was measured by qRT-PCR analysis after transfection with miRNA scrambled duplex, miR-125b mimics, miRNA inhibitor negative control and miR-125b inhibitor. Error bars represent standard deviation and were obtained from three independent experiments. [file 1476-4598-10-108-S4.TIFF]

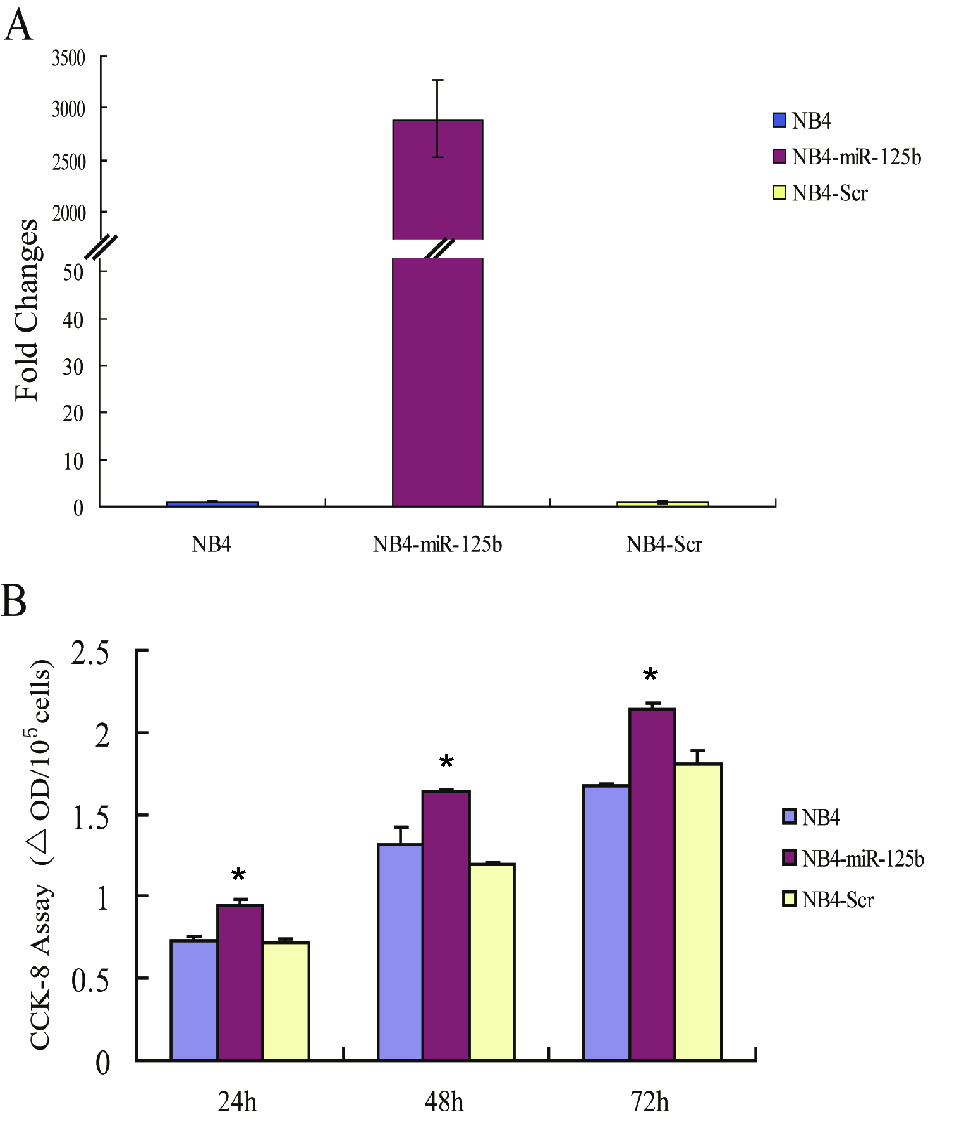

Supplement: Additional file 5 — Figure S3. miR-125b promotes cell proliferation in NB4. (A) The qRT-PCR assay was used to measure expression profiles of miR-125b. Data presented are the fold changes of miR-125b in NB4 cells transduced with lv-miR-125b and lv-Src; (B) Cell proliferation was detected using CCK-8 assay. NB4, NB4-miR-125b and NB4-Scr cells with different expression profiles of miR-125b were induced by 1 uM ATRA and cell proliferation was measured at 24 hrs, 48 hrs and 72 hrs. *p < 0.05 compared with mock (NB4) and NB4-Scr. [file 1476-4598-10-108-S5.TIFF]

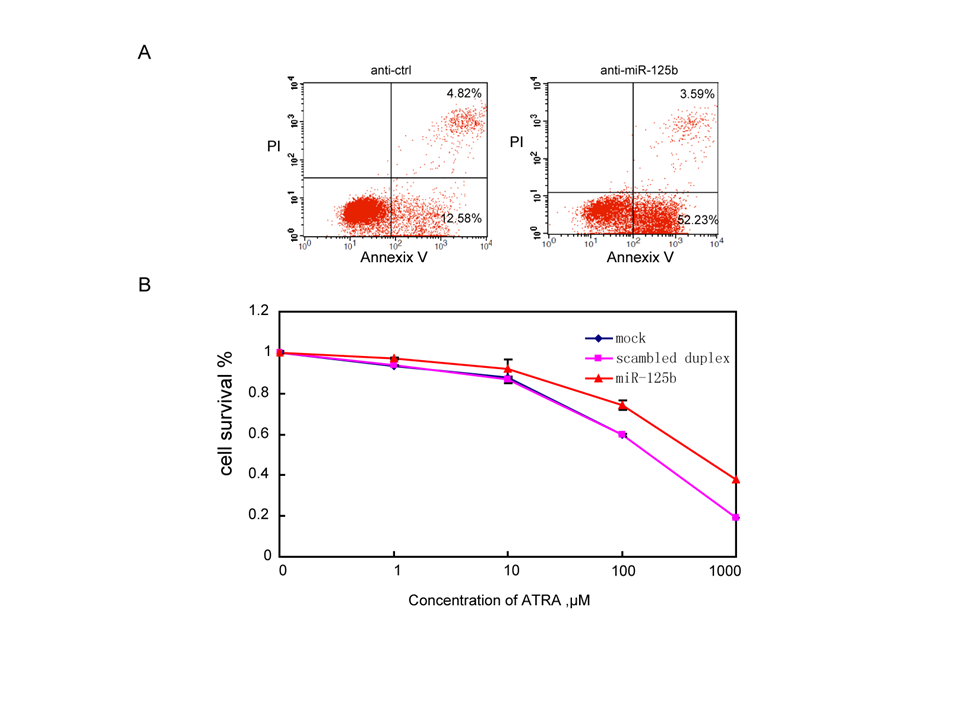

Supplement: Additional file 6 — Figure S4. miR-125b inhibits apoptosis in HL60 cells and increases NB4-R1 cells resistance to ATRA treatment. (A) The HL60 cells were transfected with 100 nM antisense control or miR-125b antisense, respectively. Forty-four hours following transfection, camptothecin was added to induce cells for four hours and then cells were labeled with Annexin V/PI and analyzed by flow cytometry. Three independent experiments were performed and similar results were obtained. (B) Transfection of NB4-R1 cells with miR-125b duplex increases their resistance to ATRA treatment. Three independent experiments were performed. [file 1476-4598-10-108-S6.TIFF]
