# Supplementary material for: Upregulation of microRNA-125b contributes to leukemogenesis and increases drug resistance in pediatric acute promyelocytic leukemia
Source: Mol Cancer. 2011 Sep 1;10:108. doi: 10.1186/1476-4598-10-108 (PMC3189170; doi:10.1186/1476-4598-10-108)
Supplement: Additional file 3 — Table S2. Primers information. [file 1476-4598-10-108-S3.DOC]

**Table S2. Primers information.**

**____________________________________________________________________**

miR-125b F 5'-CGGGATCCCCAGATACTGCGTATGTGTG-3'

miR-125b R 5'-CCGCTCGAGGTCACCTGATCCCATCTAAC-3'

BAK1-UTR-F 5'-CTAGTCTAGAGAAGATCAGCACCCTAAG-3'

BAK1-UTR-R 5'-CTAGTCTAGACTTGGAGGCTTCTGACAC-3'

BAK1-M-R 5'-CACCCCAAGCCCAGAAAGTCCAACTGCAAAGG-3'

BAK1-M-F 5'-CCTTTGCAGTTGGACTTTCTGGGCTTGGGGTG-3'

**____________________________________________________________________**

Underlined letters indicate built in restriction enzyme sites.
